# The genetic diversity and population structure of native horse breeds in Xinjiang, China

**DOI:** 10.3389/fgene.2025.1439312

**Published:** 2025-11-12

**Authors:** Chi Tang, Baoyu Yang, Gulibaheti Dawulietihan, Li Xue, Shuyuan Liu, Yinamujiang Yalimaimaiti, Qingzheng Wang, Na Yang, Xiaoyuan Sun, Yaru Wang, Ailifeire Wumaier, Serik Khizat, Tolegen Assanbayev, Zhassulan Kozhanov, Kursantbek Attokurov, Elmurat Obdunov, Hangsen Li, Aikebaier Reheman, Xiaoling Zhou, Wumaierjiang Aizimu, Kairat Iskhan, Gemingguli Muhatai

**Affiliations:** 1 College of Life Science and Technology, Tarim University, Alar, Xinjiang, China; 2 Xinjiang Production & Construction Corps Key Laboratory of Protection and Utilization of Biological Resources in Tarim Basin, Alar, Xinjiang, China; 3 Agricultural (Animal Husbandry) Development Service in Tuerhong Township, Fuyun, Xinjiang, China; 4 Animal Husbandry Workstation of Fuyun County, Fuyun, Xinjiang, China; 5 Animal Husbandry Workstation of Balikun County, Balikun, Xinjiang, China; 6 Animal Husbandry and Veterinary Station of Kalayagaqi Town, Yining, Xinjiang, China; 7 Key Laboratory of Tarim Animal Husbandry Science and Technology, Xinjiang Production & Construction Corps, Alar, Xinjiang, China; 8 Physiology, Morphology and Biochemistry, Kazakh National Agrarian Research University, Almaty, Kazakhstan; 9 Zootechnology and Veterinary Medicine, Toraighyrov University, Pavlodar, Kazakhstan; 10 Horse Breeding Department, Kazakh Research Institute of Livestock and Forage Production, Almaty, Kazakhstan; 11 Osh State University, Osh, Kyrgyzstan

**Keywords:** native horse breed, whole genome sequencing, genetic diversity, population structure, genetic resources conservation

## Abstract

**Introduction:**

Xinjiang is a region renowned for its rich diversity of native horse breeds, making it one of the most affluent equine genetic resource areas in China. While prized for their high adaptability and tolerance to roughage, the conservation of these native breeds faces challenges from the introduction of external breeds and industrial changes. Furthermore, the unknown population structure of Xinjiang horse breeds has hindered effective conservation efforts.

**Methods:**

This study presents the first comprehensive Single Nucleotide Polymorphism (SNP) analysis of seven Xinjiang native horse breeds. We utilized 10X whole-genome sequencing to assess their genetic diversity, population structure, and genetic relationships.

**Results:**

Our findings revealed a high level of population genetic diversity among the Xinjiang native horse breeds. These breeds exhibited significant genetic differentiation from other horse breeds originating from Europe, Central Asia, Western Asia, and other parts of China. Evidence of frequent historical gene flow was detected, particularly among breeds in northern Xinjiang, which were shown to be more closely related to each other.

**Discussion:**

This study elucidates the distribution patterns, evolutionary characteristics, and substantial genetic diversity of Xinjiang’s native horse breeds. The results provide crucial insights into their unique genetic background and population history. These findings offer valuable theoretical support for establishing core conservation groups of local germplasm, guiding future breeding programs for new cultivars, and further exploration of the characteristics inherent to Xinjiang’s native horse genetic resources.

## 1 Introduction

The domestic horse (*Equus ferus caballus*) was successfully domesticated approximately 5,500 years ago (Librado et al., 2021). However, recent studies have proposed that the modern domestic horse originated in the western Eurasian steppe around 4,200 years ago (Librado et al., 2021). Subsequently, they spread across Eurasia, giving rise to various breeds adapted to diverse geographic and climatic environments. Horse domestication has enhanced human work efficiency and propelled human civilization (Schubert et al., 2014). According to the Food and Agriculture Organization (FAO), there are over 900 horse breeds globally, with 694 native breeds. Among these, 138 are located in Asia and 371 in Europe (https://www.fao.org/).

The primary purposes for horse keeping include dairy and meat production, racing, equestrian activities, and maintaining free-range populations. However, agricultural advancements have decreased the overall use of horses, significantly reducing the global horse population. Over the past 2,000 years, domestic horses have lost nearly half of their genetic diversity, with genomic heterozygosity decreasing by approximately 16% in the last 200 years (Fages et al., 2019). Genetic diversity and inbreeding are often connected (Spielman et al., 2004), which can inform the efficacy of breeding conservation and offer insights into the sustainable development and utilization of horse populations. This dramatic decline in the global horse population has severely affected the genetic diversity and number of purebred native breeds. The decreased genetic diversity of domestic horses is primarily due to purebred selection practices, which increase deleterious mutations in domestic horse genomes (Orlando, 2020).

China is known for having a diverse and abundant population of horses, with a rich genetic variety (Ling et al., 2011). Furthermore, Chinese horse breeds include the Northern China (NC), Qinghai-Tibetan (QT), and Southwestern (SW) populations (Liu et al., 2019). The Xinjiang Uygur Autonomous Region (XUAR), a prominent horse-producing area in China, has the largest horse population in the country, reaching 1,107,000 horses as of 2022. This region is known for four native (Kazakh, Yanqi, Kyrgyz, Balikun) and two cultivated (Yili and Yiwu) horse breeds, mainly bred for meat. China is the largest global horse meat producer (FAO statistics, 2022), producing 159,068 tons of horse meat, most of which is produced in Xinjiang. Many horses are underutilized and freely grazed for breeding purposes, with only a small number used for meat or riding (Including leisure, competitions, celebrations, etc.). However, the demand for sports events and leisure tourism has changed the use of native horse breeds, with some regions crossbreeding native horses with Thoroughbred and Arabian breeds to achieve better competition results. This practice enhances the genetic diversity and productivity of cultivated horse breeds but significantly threatens the genetic purity of native breeds. Thus, there is a risk of extinction for native horse breeds with small population sizes due to unorganized selection and the introduction of hybrids.

Sequence analysis of mtDNA from eastern Chinese and European horse populations revealed a higher frequency of haplogroup F and a lower frequency of haplogroup D in eastern Chinese populations (Mcgahern et al., 2006). In contrast, European populations exhibited the opposite trend, suggesting a form of genetic isolation and differentiation between these two populations (Mcgahern et al., 2006). This finding was further supported by the mtDNA data from ancient horses in China dating back to 2,000–4,000 years ago (Cai et al., 2009).

Xinjiang, situated in Central Asia, the transitional region between eastern China and Europe, The ancient stone culture in Ili River basin and Boltala River basin in Xinjiang may be related to the early domestication activities (Annie and Dexin, 2020). Xinjiang Kazakh, Yanqi, and Barkun horses exhibit higher Y chromosome diversity than other horse breeds in China, possibly due to the expansive grasslands, traditional animal husbandry practices, and minimal human interference in Xinjiang (Han et al., 2015). Research on the genetic diversity and structure of Xinjiang horse breeds is limited, with most studies focusing on microsatellite markers. However, Whole Genome Sequencing (WGS) has successfully revealed the genetic diversity and structure of various horse breeds, such as the Thoroughbred (Tozaki et al., 2021), Mongolian (Huang et al., 2014), Lichuan, Kazakh (Zhang et al., 2018), and Mavari horses (Jun et al., 2014). Nonetheless, no comprehensive genome-wide study has been used to analyze the genetic information of horse breeds in Xinjiang. There is no molecular data for all the horse breeds in this region.

Therefore, this study investigated the population genetic structure of native horse breeds in Xinjiang. The study utilized WGS technology to gather genetic data from seven native horse breeds (taxa) in Xinjiang, China. The native horse breeds included Kazakh (Altay and Yili region), Yanqi, Kyrgyz, Balikun, and other unexplored breeds, such as the Kunlun (Hotan region, Kunlun Mountains) and Tashkurgan (Tashkurgan Tajik Autonomous County), These horse breeds are divided by the Tianshan Mountains, North of the Tianshan Mountains are the northern horses, which include the Kazakh and Balikun breeds, while the remaining horse breeds are distributed south of the Tianshan Mountains as southern horses. Genetic variations within and between these breeds were analyzed and compared with data from the horse breed sequences in the National Center for Biotechnology Information (NCBI; https://www.ncbi.nlm.nih.gov/). The study provides the first collection of genetic information from all Xinjiang native horse breeds. These findings will enhance our understanding of the population genetics of native horse breeds and shed light on the history of horse domestication in Xinjiang.

## 2 Materials and methods

### 2.1 Sample collection

In this study, 70 peripheral blood samples were collected from seven horse breeds (taxa), with samples taken from ten horses of each breed. The breeds included Kazakh horse in the Altay region (KA), Kazakh horse in the Yili region (KY), Yanqi (YQ), Balikun (BLK), Kyrgyz (KE), Tashkurgan (TX), and Kunlun (KL) horse. To ensure that the samples were representative of the genetic variation of the breeds, all the samples were collected from different remote pastures, with at least two separate populations sampled for each breed, and we used information provided by the owners to avoid the collection of samples from related horses and exclude parents or progeny. Herders were consulted for background information on the selection and breeding history of their native breeds. Subsequently, the genealogical records were reviewed to ensure that the samples were unrelated but representative of the genetic variation of the breeds. Blood samples from 70 domestic horses were used for Genomic DNA extraction using the TaKaRa MiniBEST Whole Blood Genomic DNA Extraction Kit (Takara Bio, Beijing, China).

The genomic DNA concentration was determined using an INVITROGEN Qubit 4.0 Fluorometer (Invitrogen Inc, CA, United States), and its integrity was assessed through agarose gel electrophoresis. Subsequently, the samples were subjected to whole-genome resequencing on the Illumina HiSeqX Ten platform (Illumina Inc, CA, United States) based on the quality of the whole-genome DNA extraction (the nucleic acid concentrations were all greater than 80 ng/μL, with OD260/280 ratios ranging from 1.8 to 2.0). The genomic DNA samples were stored at −80 °C in a freezer. In addition, sequencing data of 32 horses (including nine domestic horse breeds and one wild horse population) were downloaded from the NCBI (Supplementary Table S1). The ten horse breeds included the Akhal-Teke (n = 1), Arabian (n = 2), Curly (n = 2), Debao pony (n = 5), Mongolian (n = 4), Sorraia (n = 1), Thoroughbred (n = 4), Tibetan (n = 5), Yakut (n = 6), and Przewalski (n = 2).

### 2.2 Detection and quality control of the SNP sites

The whole genome sequences of 70 domestic horse samples yielded approximately 1831.95 GB of raw data (average of 26 GB/sample), with the Q20 and Q30 base quality exceeding 95.00% and 90.00%, respectively (Supplementary Table S1). After removing low-quality reads, each horse genome had a 10-fold coverage. The high-quality sequencing data from 70 horses, along with the sequencing data of 32 horses obtained from NCBI, were mapped to the domestic horse reference genome, EquCab3.0 (Kalbfleisch et al., 2018), Using the Burrows-Wheeler Aligner (BWA) (Li and Durbin, 2010). Eight samples with low matching rates were removed (Supplementary Table S2). Next, SAMtools v1.3 (Li et al., 2009) and GATK v3.1 (DePristo et al., 2011) were utilized to detect SNPs at variant sites. The 62 equine SNP datasets were merged with the 32 SNP datasets from the NCBI using PLINK v1.9 (Chang et al., 2015). Subsequently, SNP loci with <90% detection rates, a <0.05 minor allele frequency (MAF), and >1% missing genotype rates were filtered out using pi-hat in PLINK v1.9 (Chang et al., 2015), and the pedigree relationships between samples were evaluated (Petersen et al., 2013). The filter parameters were as follows: (1) Fisher test of strand bias (FS) ≤ 60; (2) HaplotypeScore ≤13.0; (3) Mapping Quality (MQ) ≥ 40; (4) Quality Depth (QD) ≥ 2; (5) ReadPosRankSum ≥ −8.0; and (6) MQRankSum > −12.5 (Supplementary Table S3; Supplementary Figures S1–S3). Linkage disequilibrium (LD) was evaluated by calculating r^2^ at the genome-wide intervals of 25 SNPs with a window size of 100 SNPs. The dataset was further filtered to remove samples with low quantities, retaining others representative of the five horse breeds, using a 0.2 threshold for judging LD (--indep-pairwise 100 25 0.2).

### 2.3 Analysis of genetic diversity and population structure

The expected heterozygosity (He) and coefficient of inbreeding (Fis) were calculated using the PLINK software. The He for each population was performed through the Hardy-Weinberg test, while Fis was calculated per sample using the number of pure heterozygotes. Furthermore, the principal component analysis (PCA) and population differentiation index (Fst) were determined using smartPCA in the EIGENSOFT software v.4.2 (Price et al., 2006), The proportion of missing data to be ≤10% was allowed. Phylogenetic trees were constructed using the neighbor-joining (NJ) method in FastTree v.2.1.11 (Price et al., 2009), The resulting tree file was subsequently uploaded to the iTol v5 (Letunic and Bork, 2021) for visualization purposes. Phylogenetic networks were visualized in R version 3.5.1. The genetic structure of the population was assessed with the fastSTRUCTURE package (Raj et al., 2014). The results of the fastSTRUCTURE analysis were then visualized using the DISTRUCT software (Rosenberg et al., 2002).

## 3 Results

**FIGURE 1:**
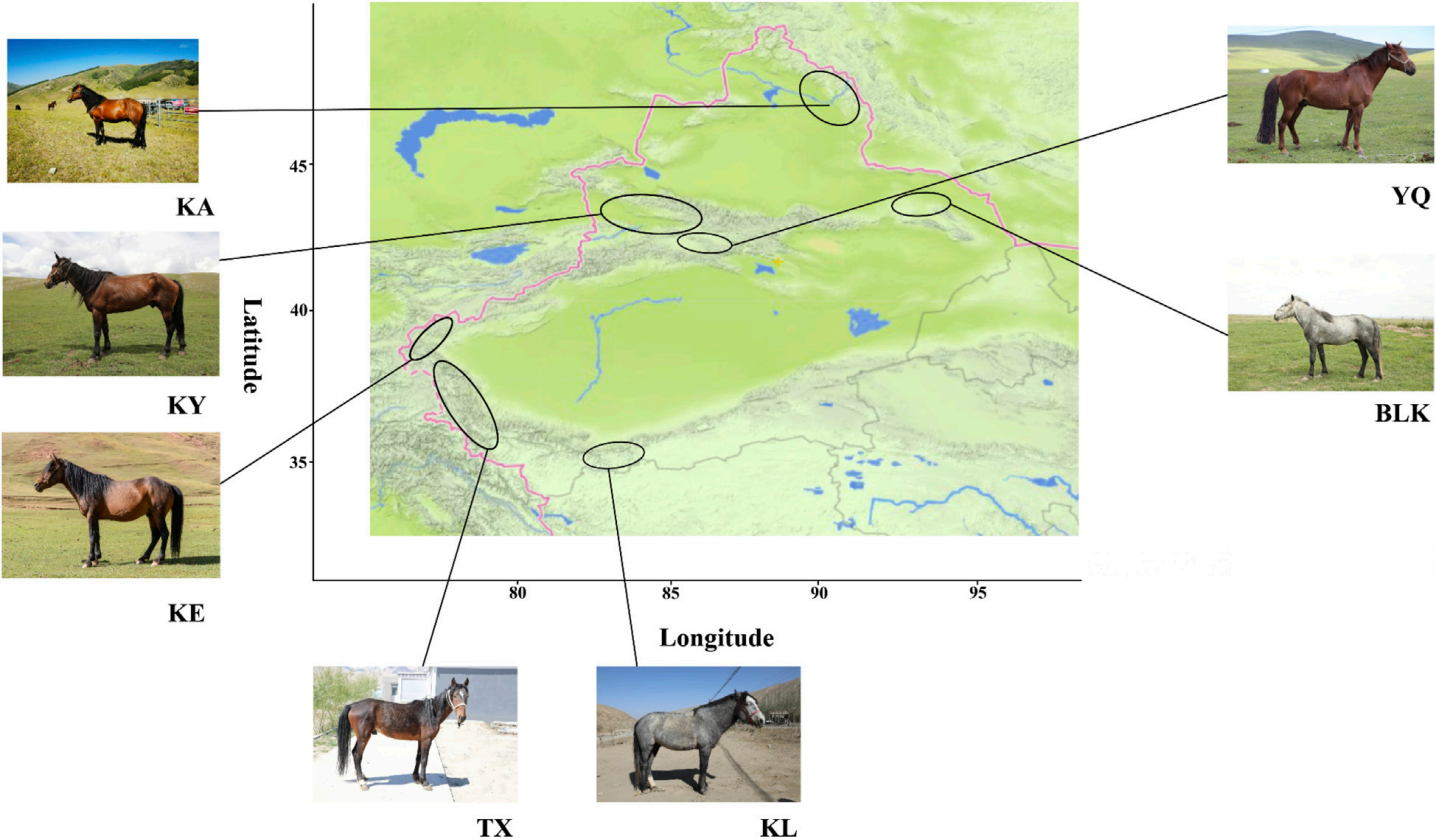
Map of the native horse breed distribution in Xinjiang. KA, Kazakh horse in the Altay region; KY, Kazakh horse in the Yili region; YQ, Yanqi horse; BLK, Balikun horse; KE, Kyrgyz horse; TX, Tashkurgan horse; KL, Kunlun horse.

The four main Xinjiang horse breeds, developed in diverse environmental conditions, exhibited distinct characteristics (Figure 1). The 94 samples that met the specified quality control criteria generated 26,539,717 SNPs (Supplementary Table S4).

### 3.1 Analysis of genetic diversity

Table 1 shows that the heterozygosity values across all the breeds and populations were notably similar. The observed heterozygosity (Obs Het) reflects the actual proportion of heterozygosity within the population, The genetic diversity of the Xinjiang native horse breeds was found to be relatively low compared to Mongolian and Tibetan horse (Supplementary Table S5). Furthermore, the Obs Het for all the populations was lower than the expected heterozygosity (Exp Het), suggesting the presence of varying degrees of inbreeding or genetic drift. Additionally, Fisher’s inbreeding coefficient (Fis) provided further insights into the degree of inbreeding; notably, the YQ population exhibited the highest Fis value (0.1844). The Fis results for the Xinjiang native horse breeds indicated generally low levels of inbreeding, with the lowest coefficients observed in the KE and KL populations, likely attributed to differences in stallion distributions within these groups. The polymorphic information content (PIC/Pi) value of 0.2689 indicates that the KL population exhibits higher levels of polymorphism and more pronounced genetic differences among individuals. This elevated Pi value reflects a greater genetic diversity, which is crucial for the adaptation of the assessment population. Understanding this genetic diversity is significant for breeding strategies and overall population management.

**TABLE 1:** Comparison of genetic diversity of Xinjiang horse breeds.

Breed/Population	Obs Het	Exp Het	Pi	Fis
YQ	0.1940	0.2481	0.2632	0.1844
BLK	0.2207	0.2541	0.2682	0.1336
KY	0.2330	0.2543	0.2697	0.1013
KE	0.2294	0.2486	0.2619	0.0915
TX	0.2248	0.2472	0.2668	0.1061
KL	0.2408	0.2536	0.2689	0.0781
KA	0.2285	0.2490	0.2693	0.1026

**FIGURE 2:**
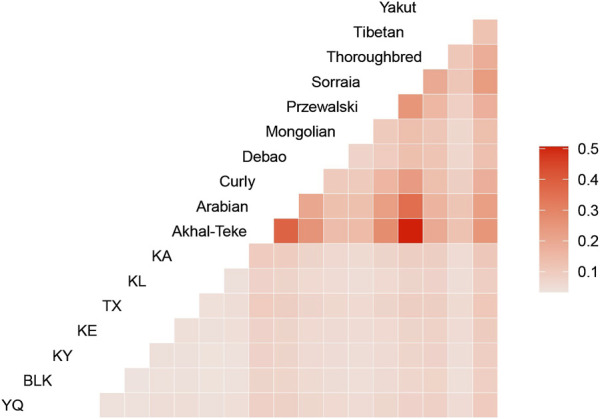
Heatmap illustrating genetic differentiation among the horse breeds, with population differentiation (FST) values ranging from 0.1 to 0.5, indicated by a gradient from light to dark red.

The genetic differentiation index (Fst) between the horse groups demonstrates the genetic divergence among the different horse populations. The differentiation within the Xinjiang native horse breeds was relatively lower than that of the other breeds. The Fst values for the Xinjiang horse breeds did not surpass 0.01, indicating minimal differentiation among the samples. In contrast, the Arabian and Akhal-Teke horses exhibited higher differentiation from the remaining groups, with the highest Fst compared with the other horse breeds (Figure 2). Although Przewalski’s horses diverged from domestic horses approximately 40,000 years ago, their Fst values with domestic breeds were lower than expected (Figure 2). This pattern may reflect shared ancestral polymorphisms at conserved neutral loci, as captured by the SNP array. Additionally, demographic histories, such as bottlenecks during domestication, may have contributed to the homogenization of diversity at certain loci. This may be related to their breeding strategies. Both horse breeds have undergone long-term closed breeding and intensive selection to preserve their unique and exceptional traits, such as the endurance and elegance of Arabian horses, as well as the speed and endurance of Akhal-Teke horses. While these practices maintain the desired breeding characteristics, they also result in a decrease in genetic diversity and an increase in interpopulation differentiation.

**FIGURE 3:**
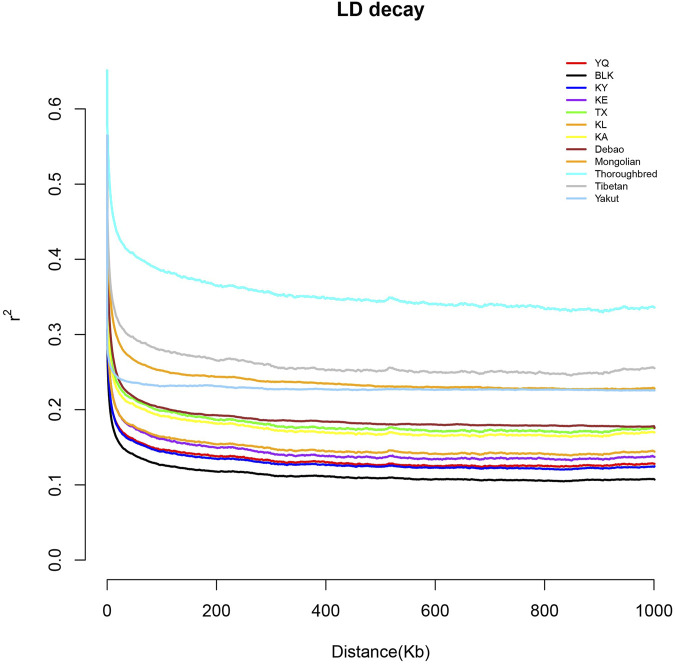
Linkage disequilibrium decay of the studied horse breeds.

The Xinjiang native horse breeds had consistently lower LD Decay rates than the domestic native and foreign breeds (Figure 3), suggesting a higher genetic diversity than non-Chinese breeds retrieved from NCBI, with BLK having the highest value in this study. In contrast, Thoroughbreds, having undergone extensive and systematic selective breeding and controlled reproduction to meet specific performance requirements, especially for racing, had the lowest genetic diversity.

### 3.2 Genetic structure

**FIGURE 4:**
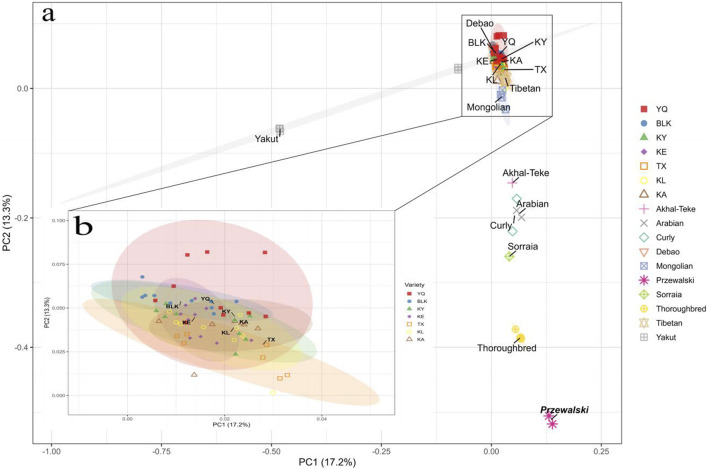
Principal component analysis of the first two components of **(a)** all the horse samples and **(b)** all the Xinjiang native horses. The ellipses indicate the boundaries for the points corresponding to the breeds, with a confidence level of 0.95. The positions of the breed labels correspond to the central points.

The PCA revealed limited differentiation among the Xinjiang native horse breeds. These native breeds clustered together with considerable overlap (Figure 4). The results of the Principal Component Analysis (PCA) presented in Figure 4A demonstrate a distinct separation between the native horse breeds of China and the foreign horse breeds, utilizing Przewalski’s horse as the reference population. Among them, the Yakut, Throughbred, Sorraia, and Curly horse breeds were obviously different from Xinjiang native horse breeds, while Throughbred and Yakut showing notable separation from the other horse breeds. Figure 4B shows the distribution of these Xinjiang native horse breeds on principal component PC1 and PC2, with different colored ellipses indicating the 95% confidence intervals for each population. The size and shape of these ellipses reflect the genetic differences between the populations. Among them, the confidence ellipses of KA and KE were smaller and overlapping, indicating that they had a similar genetic background. The Xinjiang native horse breeds are closest in genetic background to Mongolian and Tibetan horses, possibly due to the historical exchanges between these breeds. Furthermore, the PCA results revealed significant genetic differentiation between the Central and Western Asian breeds, including Arabian and Akhal-Teke horses, corroborating the Fst results.

**FIGURE 5:**
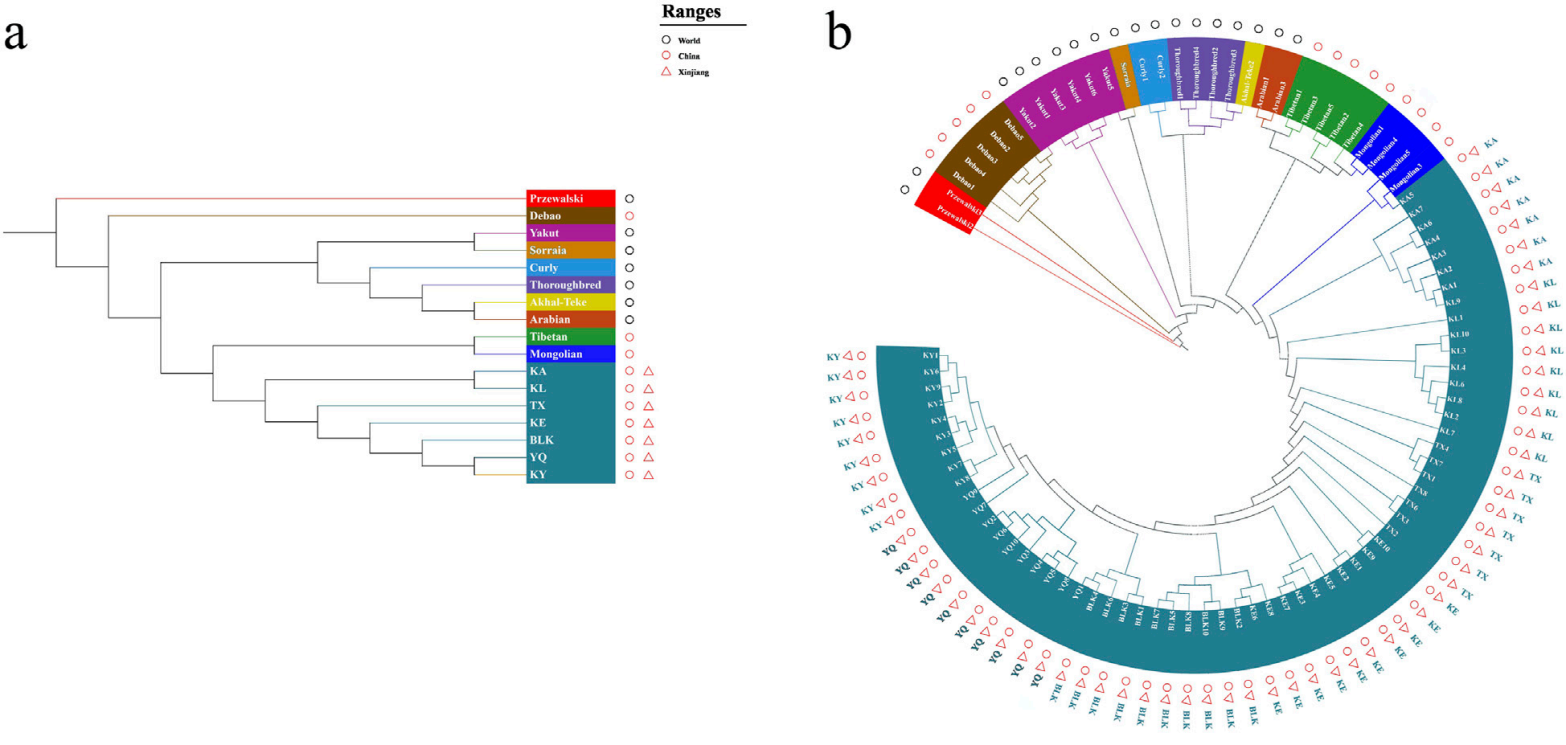
The Neighbor-Joining tree of the horse breeds. The bootstrap value was close to 100%. **(a)** Breeds, **(b)** individuals.

The native horses of Xinjiang were classified into a single large taxon, indicating that these horses are largely distinct from non-Xinjiang breeds. This pattern is more likely due to the long-term independent evolutionary history of Xinjiang horses, which has led to the accumulation of unique mutations over generations, rather than solely due to unique genetic characteristics during early domestication. (Figure 5A). Moreover, the Debao horse, which exhibits a unique evolutionary pattern characterized by a distinct genetic lineage, stands out from other breeds. Meanwhile, the remaining Chinese and foreign horse breeds can be broadly categorized into two major genetic clusters. Subsequent classification identified two main groups in southern and northern Xinjiang, highlighting a significant genetic distance between the breeds in these regions and reflecting the geographic distance (Figure 5B). The Debao pony exhibited the most primitive branching, while the Mongolian and Tibetan horses displayed a closer phylogenetic relationship. In Xinjiang, KA, KY, YQ, and BLK were closely related to the northern Xinjiang breeds, whereas KE and TX were more closely related to the southern Xinjiang breeds, which is consistent with the findings of the PCA results.

**FIGURE 6:**
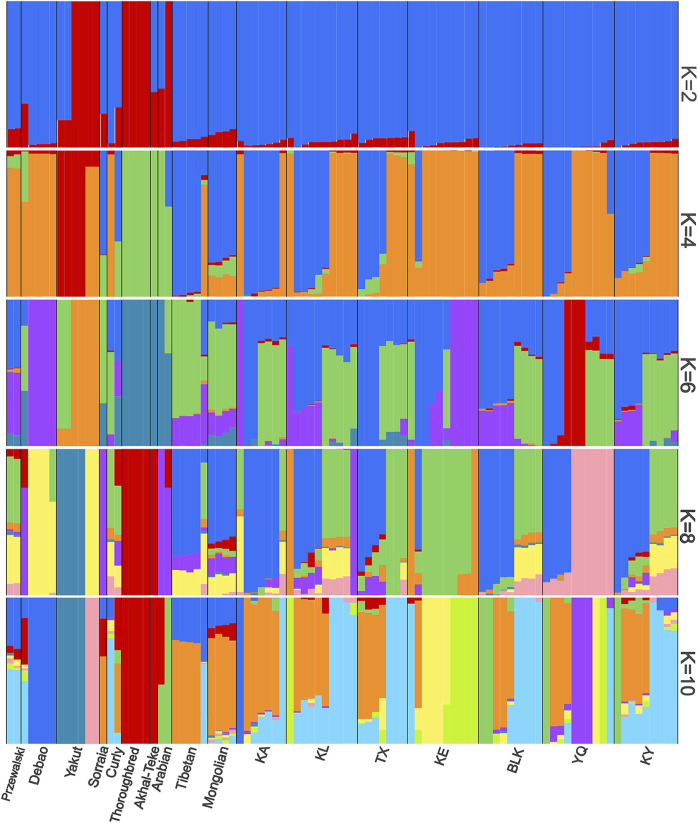
Comparative structure of the horse populations with the number of ancestral clusters (K) ranging from 2 to 10. Each bar represents an individual for each breed.

The population structure was analyzed for all the horse samples, considering a K value between 2 and 10, representing the ancestral groups (Figure 6). At a value of K = 4, we could distinguish the separate groups of horse breeds in China and horse breeds in other countries (Supplementary Figure S4). The population genetic structure analysis revealed that at K = 6, the Xinjiang native horse breeds were distinct. The analysis of K = 10 reveals that the genetic structure of the native horse breed in Xinjiang is highly complex. This complexity arises not only from random genetic drift or mutations but is more likely attributable to prolonged gene flow.

## 4 Discussion

This study included seven Xinjiang native (KY, YQ, BLK, KE, TX, and KL), three Chinese (Mongolian, Tibetan, and Debao pony), and six foreign (Thoroughbred, Curly, Yakut, Sorraia, Arabian, and Akhal-Teke) horse breeds. Xinjiang native horse breeds exhibited typical genetic characteristics that low inbreeding levels, aligning with the traditional open breeding practices on Xinjiang grasslands. This result suggests strong resistance to inbreeding within this population. Furthermore, Thoroughbreds had the lowest genetic diversity due to varying degrees of bottleneck effects caused by closed artificial selection and high levels of inbreeding (Khanshour et al., 2013; Cosgrove et al., 2020). Ethnic groups in Xinjiang prefer specific horse coat colors; for example, Kazakhs favor dark colors (red bay and black bay), and Tajiks favor light color horses. Therefore, selections are based on coat color preferences. Hence, Thoroughbred and Arabian crosses with desired coat colors were introduced. Interestingly, the genetic structure, which was influenced by coat color preferences, has maintained the genetic diversity of the horse breeds (Druml et al., 2009).

Xinjiang horse breeds are genetically distinct from other Chinese, Central Asian, and European horse breeds, This differentiation is likely primarily driven by the long-term independent evolutionary history following the divergence of these horse breeds from other populations. Additionally, other factors such as historical population bottleneck effects, genetic drift, special environmental adaptability, and targeted artificial selection for specific traits (Castaneda et al., 2019) may have further contributed to their genetic distinctiveness. Moreover, Xinjiang horse breeds have a relatively independent genetic structure and are more closely related to Tibetan and Mongolian horses. The genetic makeup of Xinjiang horse breeds, particularly Kazakh horses, aligns with previous research findings (Liu et al., 2019). Crossbreeding practices in Xinjiang involve using native horse breeds as dams and Arabian or Thoroughbred horses as sires to produce offspring with enhanced speed and endurance for competitive events. Thus, this study included all purebred native breeds, suggesting that other breeds were introduced in the past for crossbreeding to improve performance, albeit with challenges in tracing their pedigree and genealogical information due to insufficient records. This study, based on a genetic analysis of 32 horses from outside Xinjiang using genome-wide autosomal SNPs, revealed significant genetic distinctions, reflecting their geographic origin and breed history (Petersen et al., 2013). Conversely, studies on the Kazakh horse mtDNA indicated that native horse breeds in western China have multiple matrilineal origins and exchange genetic material with each other and other horse breeds during mobility (Zhang et al., 2012). The PCA and structural results from this study indicate that Kazakhstan possesses a complex genetic background, with both autosomal and mitochondrial DNA (Gemingguli et al., 2016) findings corroborating each other.

Historical interbreeding probably influenced horse breeds divergence. Archaeological data revealed significant gene flow between populations in the Pamir Plateau and Ferghana Valley, acting as a ‘transmitter’ for exchanges between eastern (Tarim Basin, China) and western (Bactria, Uzbekistan) populations (Hemphill and Mallory, 2004). Horse breeds in the Middle East are closely related to local horse breeds in Xinjiang and may be influenced by human activities. Future research should investigate the gene flow between these populations in conjunction with historical events.

In our analysis, we observed distinct genetic clusters among different horse breeds, which highlights the genetic differentiation between breeds. However, it is important to note that within each breed, there may exist multiple distinct subgroups with significant genetic differences. This intrabreed heterogeneity could be due to factors such as regional differences, selective breeding practices, or historical gene flow events. Genetic differentiation has been observed within various native horse breeds in Xinjiang, particularly in the Northern Xinjiang region. This differentiation is primarily attributed to the introduction of foreign germplasm for crossbreeding. Since herders often introduced Thoroughbreds for crossbreeding, this could seriously affect the purebred germplasm resources and reduce the genetic diversity of the native horse population in Xinjiang.

## 5 Conclusions

This study presents the first comprehensive analysis of whole genome sequences of Xinjiang native horse breeds, revealing the genetic diversity and structure of these breeds. Xinjiang native horse breeds exhibited higher genetic diversity, with evidence of gene exchange and similarities in the genetic backgrounds of the different groups. The genetic structure of the southern and northern border breeds differed. Additionally, Tashkurgan and Kunlun horses may represent new cryptic horse breeds. Phylogenetic analysis shows that Tashkurgan and Kunlun horses form a distinct clade separate from other Xinjiang breeds (see Figure 5). These horses also exhibit unique genetic signatures in their SNP profiles, indicating a degree of genetic isolation and differentiation. Further investigation is warranted to fully characterize these potential cryptic breeds. Our findings assessed genetic variability and inbreeding within these horse breeds, thereby laying a foundation for future horse breeding policies. They could inform the delineation of protected areas and breeding grounds to prevent large-scale inbreeding and hybridization and will provide a valuable reference for the conservation and utilization of native horse breeds in Xinjiang.

## Data Availability

The data presented in the study are deposited in the NCBI SRA repository, accession number PRJNA1185891.
